# Initial Variability and Time-Dependent Changes of Neuronal Response Features Are Cell-Type-Specific

**DOI:** 10.3389/fncel.2022.858221

**Published:** 2022-04-27

**Authors:** Jens-Steffen Scherer, Oda E. Riedesel, Ihor Arkhypchuk, Sonja Meiser, Jutta Kretzberg

**Affiliations:** ^1^Computational Neuroscience, Department of Neuroscience, Faculty VI, University of Oldenburg, Oldenburg, Germany; ^2^Cluster of Excellence Hearing4all, Department of Neuroscience, Faculty VI, University of Oldenburg, Oldenburg, Germany; ^3^Research Center Neurosensory Science, Carl von Ossietzky Universität Oldenburg, Oldenburg, Germany

**Keywords:** invertebrate, leech, mechanoreceptor, touch cell, pressure cell, Retzius cell, spike count, resting membrane potential

## Abstract

Different cell types are commonly defined by their distinct response features. But several studies proved substantial variability between cells of the same type, suggesting rather the appraisal of response feature distributions than a limitation to “typical” responses. Moreover, there is growing evidence that time-dependent changes of response features contribute to robust and functional network output in many neuronal systems. The individually characterized Touch (T), Pressure (P), and Retzius (Rz) cells in the medicinal leech allow for a rigid analysis of response features, elucidating differences between and variability within cell types, as well as their changes over time. The initial responses of T and P cells to somatic current injection cover a wide range of spike counts, and their first spike is generated with a high temporal precision after a short latency. In contrast, all Rz cells elicit very similar low spike counts with variable, long latencies. During prolonged electrical stimulation the resting membrane potential of all three cell types hyperpolarizes. At the same time, Rz cells reduce their spiking activity as expected for a departure from the spike threshold. In contrast, both mechanoreceptor types increase their spike counts during repeated stimulation, consistent with previous findings in T cells. A control experiment reveals that neither a massive current stimulation nor the hyperpolarization of the membrane potential is necessary for the mechanoreceptors’ increase in excitability over time. These findings challenge the previously proposed involvement of slow K^+^-channels in the time-dependent activity changes. We also find no indication for a run-down of HCN channels over time, and a rigid statistical analysis contradicts several potential experimental confounders as the basis of the observed variability. We conclude that the time-dependent change in excitability of T and P cells could indicate a cell-type-specific shift between different spiking regimes, which also could explain the high variability in the initial responses. The underlying mechanism needs to be further investigated in more naturalistic experimental situations to disentangle the effects of varying membrane properties versus network interactions. They will show if variability in individual response features serves as flexible adaptation to behavioral contexts rather than just “randomness”.

## Introduction

In the last two decades, an increasing body of literature has come to appreciate neuronal variability as flexibility rather than instability and noise of nervous systems (Prinz et al., 2004; Stein et al., 2005; Marder, 2011; Waschke et al., 2021). Single cells and networks of cells possess unique, yet functional, configurations for their membrane properties in many vertebrates and invertebrates. Examples can be found in a huge variety of systems, including the stomatogastric ganglion of lobsters and crabs (Golowasch et al., 2002; Prinz et al., 2004; Bucher et al., 2005; Marder and Goaillard, 2006; Marder, 2011; Calabrese, 2017), the central pattern generator of the leech heart beat (Calabrese et al., 2011; Norris et al., 2011; Roffman et al., 2012; Marin et al., 2013; Wenning et al., 2018), the visual system of drosophila (Linneweber et al., 2020), neurons of the pre-cerebellar nucleus Area II of goldfish (Weaver and Wearne, 2008), cerebellar Purkinje neurons (Swensen and Bean, 2005), stellate cells in the medial entorhinal cortex of mice (Pastoll et al., 2020), and globus pallidus and hippocampal neurons in rats (Günay et al., 2008; Rathour and Narayanan, 2019).

Most vertebrate systems require more complex experimental approaches like cell labeling (Baier and Scott, 2009) for the unambiguous classification of cell types. In contrast, many invertebrate systems including the stomatogastric ganglion in lobsters and crabs (Marder and Bucher, 2007), the fly visual system (Borst and Haag, 2002), *C. elegans* (Hall and Russell, 1991), and the leech ganglion (Wagenaar, 2015) allow the individual cell characterization based on anatomical properties like the position of the cell body, electrophysiological features like characteristic shapes of spikes, or their characteristic responses to specific sensory or current stimuli. Therefore, invertebrate systems are particularly well suited for the analysis of individual differences between cells of the same type (Prinz et al., 2004), as well as the investigation of fundamental mechanisms of neuronal flexibility, such as homeostatic synaptic plasticity (Turrigiano and Nelson, 2004; Marder and Goaillard, 2006; O’Leary et al., 2014; Städele and Stein, 2016), and neuromodulation (Burrell et al., 2001; Kandel, 2005; Kristan et al., 2005; Shomrat et al., 2010; Marder, 2012; Temporal et al., 2012).

One of these classic invertebrate model organisms is the medicinal leech (Tomina and Wagenaar, 2017). Each of its segmental ganglia contains only ∼400 large, well-characterized, easily accessible neurons, allowing electrophysiological investigation of specific cell types and their functions across preparations (Kristan et al., 2005; Wagenaar, 2015). Despite its comparatively small nervous system, leeches can discriminate touch locations on their skin similarly well as the human fingertip (Thomson and Kristan, 2006; Pirschel and Kretzberg, 2016). Tactile input to the skin is processed by three different mechanosensory cells: touch (T) cells, pressure (P) cells and nociceptive (N) cells. Each segmental ganglion comprises two bilateral pairs of both P and N cells (Burrell, 2017), which cover either the ventral or dorsal skin area on one side. Additionally, there are three bilateral T cells within each side of the ganglion whose processes reach into the lateral, ventral, and dorsal skin regions (Nicholls and Baylor, 1968). To minimize the potential variability within the samples of T cells and of P cells, we restricted our study to the subtypes T3 cells and P1 cells, according to their anatomical soma position in the ganglion (Tomina and Wagenaar, 2017, see their Figure 1C). Since there are no known physiological differences between the members of bilateral neurons in each ganglion (Hagiwara and Morita, 1962), we did not distinguish between the left and the right Rz, T3, and P1 cells. Moreover, the mechanoreceptors are laterally coupled. T cells are coupled to each other (Baylor and Nicholls, 1969a) and receive polysynaptic input from the P and N cells, presenting a combination of excitatory and inhibitory potentials (Burgin and Szczupak, 2003).

Recently, Meiser et al. (2019) found that repeated somatic stimulation in T cells led to a hyperpolarization of the resting membrane potential over time in addition to an increased spike count. Modeling results suggested a two-step mechanism of short-term intrinsic plasticity: First, repeated stimulation leads to Na^+^ influx and therefore to Na^+^/K^+^ pump activation, slowly hyperpolarizing the resting membrane potential. Second, hyperpolarization closes putative slow, voltage-dependent K^+^-channels, mitigating K^+^ efflux and therefore causing increased spiking in response to a given current pulse.

In the present study we set these time-dependent changes of neuronal response features in the context of variability between individual cells of three different types. Besides the mechanosensory T cell, we included the P cell as a further mechanoreceptor and the Retzius (Rz) cell as a key neuromodulatory cell that releases serotonin in the leech nervous system (Lent et al., 1979; Leake, 1986; Sahley, 1995; Burrell et al., 2001; De-Miguel et al., 2015) and possesses a different set of membrane ion channels (Kleinhaus and Prichard, 1983; Valkanov and Boev, 1988; Stewart et al., 1989; Johansen, 1991; Gerard et al., 2012; Kim et al., 2019; Angstadt et al., 2021; Heath-Heckman et al., 2021).

For each of these cell types, we investigated the variability of the resting membrane potential, the spike count, and the latency in response to current stimulation, as well as the occurrence of rebound spikes after hyperpolarization. In addition to the expected distinction between the response features of the three cell types, we also found their variability as well as their time-dependent changes to be cell-type-specific.

## Materials and Methods

### Animals and Preparation

We performed the experiments on in total 78 adult hermaphrodite medicinal leeches (hirudo verbana; *Biebertaler Leech Breeding Farm*, 35444 Biebertal, Germany), kept at room temperature in 24 L tanks filled with artificial pond water (ocean sea salt diluted with purified water, 1:1,000) under natural day-light-cycles. Before and during dissection, we anesthetized the leeches in ice-cold leech saline (115 mM NaCl, 4 mM KCl, 1.8 mM CaCl_2_, 10 mM Glucose, 4.6 mM Tris–maleate, 5.4 mM Tris base; pH 7.4; Muller and Scott, 1981). Isolated midbody ganglia 7–16 were dissected and pinned ventral side up in a petri dish with silicone elastomer *Sylgard* (Dow Corning Corporation, Midland, MI, United States). To avoid potential effects of the stimulus history and of network inputs from previously stimulated cells, we only performed one experiment per ganglion.

### Electrophysiological Techniques

We performed intracellular single recordings on the mechanosensory T3 and P1 cell (Tomina and Wagenaar, 2017, their Figure 1C), as well as on the neurosecretory Retzius cell (Rz) using sharp electrodes filled with 4 M potassium acetate (pH adjusted to 7.4). Electrode resistances ranged from 10 to 26 MΩ (mean = 17.58 MΩ, std = 2.59 MΩ). Electrodes were pulled from borosilicate microelectrodes (TW100F-4, World Precision Instruments Inc., Sarasota, FL, United States) with a P97 Flaming Brown micropipette puller (Sutter Instruments Company, Novato, CA, United States). We performed the recordings using a mechanical micromanipulator type MX-1 (TR 1, Narishige, Tokyo, Japan) and a SEC-05X amplifier (setup 1, experimenter IA) or BA-1s amplifier (setup 2, experimenter OR) (NPI Electronic, Tamm, Germany). We identified the three cell types according to their soma location and response patterns (Nicholls and Baylor, 1968). Current was injected into the soma while recording the membrane potential (sample rate 10 kHz; custom MATLAB software (R2021b), MathWorks, Natick, MA, United States). During the T cell recordings, we additionally tracked the temperature in the petri dish using a digital multimeter (PeakTech 2025, Ahrensburg, Germany).

### Experimental Design

We used two different protocols in this study, a stimulation protocol, and a control protocol. Both protocols were repeatedly presented up to a total duration of 10 min.

**FIGURE 1:**
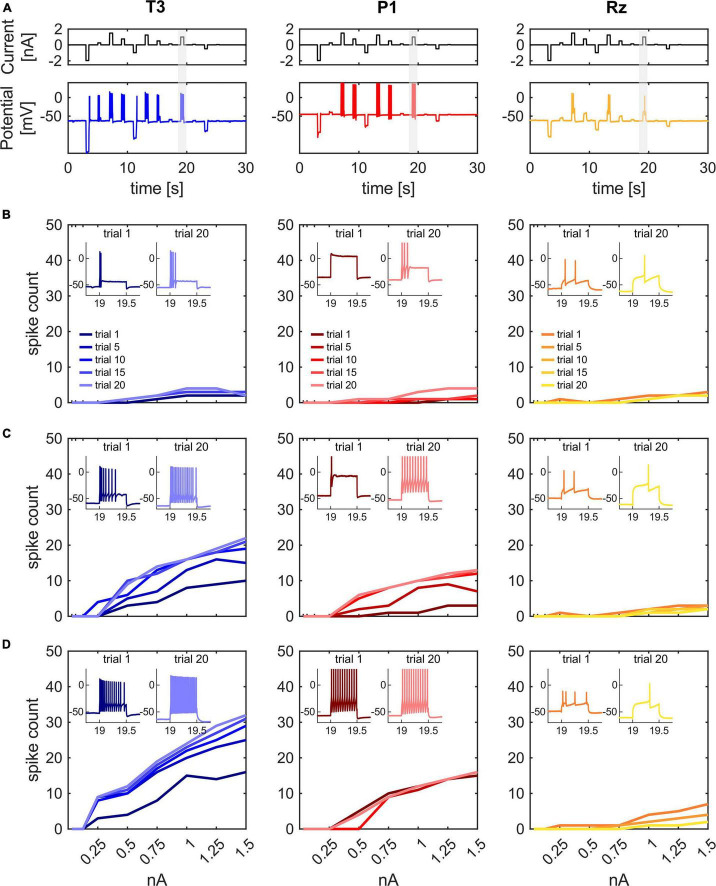
Stimulus protocol with exemplary recordings and fI curves for T, P and Rz. **(A)** The stimulus protocol used in this study, consisting of pseudo-randomized step currents applied intracellularly. Example recordings of a typical T3 (blue), P1 (red) and Rz (yellow) cell at trial 10 (after 5 min). The gray bar highlights the 500 ms long 1 nA step current, which is taken as representative response in the following analyses. **(B–D)** fI-curves for three different exemplary cells of each cell type in trials 1, 5, 10, 15, and 20 (earlier trials are shown in darker colors). The y-axis shows the absolute numbers of spikes during the step currents of 500 ms each. Insets show the responses to the 1 nA current pulse in trials 1 (at the start of the recording) and 20 (after 10 min) with recording time in seconds on the x-axis and membrane potential in mV on the y-axis.

– Stimulation protocol (stim): To replicate our previous findings, we stimulated single T3, P1 or Rz cells with the stimulus protocol developed by Meiser et al. (2019). In brief, the stimulation has a total length of 30 s and consists of pseudo-randomized current pulses, varying in amplitude from −2 to + 1.5 nA, with a duration of 500 ms each, separated by 1,500 ms without current input (Figure 1A). We recorded 20 trials to compare the initial response properties with the responses after 10 min.

– Control protocol (cont): To investigate if the changes in activity observed in our study depend on previous spiking activity and to detect synaptic inputs, we used a control protocol consisting of two step currents of −0.25 and + 1 nA with a duration of 500 ms each, followed by 5 min of no stimulation. Since measurements were taken at the beginning of each trial, we recorded three trials to compare the initial responses with the responses after 10 min.

### Data Analysis

We analyzed a total of *n* = 180 recordings. The stimulation protocol was applied to: T3 cell (*n* = 67), P1 cell (*n* = 29), Rz cell (*n* = 21); and the control protocol to: T3 cell (*n* = 19), P1 cell (*n* = 22), Rz cell (*n* = 22). The following response features were assessed as dependent variables for the statistical analysis:

– RMP [resting membrane potential, (mV)] was defined as the median membrane potential calculated over 1 s immediately before the 1 nA current pulse. Note that in the stimulation protocol the RMP was measured after several current steps were applied (second 18.0–19.0), while in the control protocol it was determined at the beginning of the recording (second 4.5–5.5).
– Spike count was defined as the total number of spikes elicited by a neuron during the 500 ms current pulse of +1 nA. Spikes were reliably detected using the *findpeaks*-function in MATLAB R2021b (parameter settings: *MinPeakHeight* = −20, *MinPeakProminence* = 10, *MinPeakDistance* = 50). We only included cells that spiked at least once in response to the 1 nA current pulse in the first or last trial.
– Rebound spike count was defined as the number of spikes elicited by a neuron in the 500 ms time window immediately after the −2 nA current pulse in the stimulation protocol. Rebound spikes could not be determined for the control protocol, which did not contain a strong hyperpolarization.
– Latency (ms) was defined as the period between the start of the 1 nA current pulse and the peak of the first spike, as determined by the *findpeaks*-function.
– ISI [inter-spike-interval, (ms)] was defined as the period between the peaks of two spikes. To receive meaningful distributions, we pooled all ISIs over all trials.
– Voltage sag ratio was defined as the ratio between the voltage sag amplitude and the passive response during the −2 nA current pulse. The passive response (mV) was defined as the potential difference between the median values of the membrane potential during the last 50 ms of the −2 nA current pulse and the last 500 ms before the onset of this current pulse. Accordingly, the voltage sag amplitude (mV) was calculated as the difference between the local minimum of the membrane potential and the median of the membrane potential during the last 50 ms of the −2 nA current pulse. The voltage sag ratio was calculated for all cell types in the stimulation protocol.
– Input resistance (MΩ) was defined as the absolute value of the passive response as defined before, divided by −2 nA. The input resistance was calculated for all cell types in the stimulation protocol.

For comparisons over time, we measured the first four response features in the first trial (“initial”) and after 10 min in the last trial (“final”). The difference of the final response and the initial response is indicated by the symbol Δ (i.e., trial 20—trial 1 for the stimulation protocol, trial 3—trial 1 for the control protocol). We corrected all final RMP values for their individual, mostly negative electrode offset (mean = −2.66 mV, std = 3.37 mV) after the experiment by assuming a linear electrode drift over time.

To further investigate the origins of the variability in response properties in T3 cells, we analyzed the influence of the following four confounder variables on their initial spike count:

– Temperature (°C) was defined as the temperature in the petri dish measured at the beginning of each experiment, ranging from 21.5 to 24.6°C. Since leeches are cold-blooded animals, their body temperature heavily depends on the temperature in the environment, which in turn can have drastic effects on the nervous system and its activity (Angstadt and Calabrese, 1989; Catarsi and Brunelli, 1991a; Hitchcock et al., 2017).
– Leech individual (ID) was defined as unique ID assigned to leech individuals.
– Ganglion (number) was defined as the number of the respective mid-body ganglion counting from anterior to posterior, ranging from 7 to 16.
– Experimenter/Setup (ID) was defined as a unique ID assigned to each of the two experimenters that collected the data (IA or OR). Both used comparable experimental equipment but in different rooms. Hence the ID IA also refers to the experimental setup 1, and OR refers to setup 2.

We used the coefficient of variation (*CV*) to characterize the dispersion in the initial spike counts, initial latencies and ISIs. Since the collected data and their measures did not follow a normal distribution, we chose non-parametric tests for the statistical evaluation of our results. We calculated Spearman’s rank correlations (*r*_*s*_) against zero to determine statistical significance (α = 0.05). Correlations deviating significantly from zero are marked in bold (Figures 3A,C, 4, 5). Direct comparisons between the median of two distributions were accomplished using the non-parametric Wilcoxon rank sum test (Figures 3D, 6). Deviations from zero were tested using the Wilcoxon signed-rank test (Figures 6C,F; Wilcoxon, 1945). Since datasets were partially used for multiple testing (Figure 6), we corrected the significance level in these cases using the Bonferroni method (α’ = α/5 = 0.01). All statistical tests of this study are summarized in Table 1. For α = 0.05, all values are rounded up to the second decimal place and *p*-values below 0.01 are indicated as < 0.01. For α’ = 0.01, all values are rounded up to the third decimal place and *p*-values below 0.001 are indicated as < 0.001.

**TABLE 1:** Interdependencies among outcome variables in T3 and P1 and Rz cells.

Spearman correlations		T3			P1			Rz		
x	y	*n*	*r*_*s*_	*p*	*n*	*r*_*s*_	*p*	*n*	*r*_*s*_	*p*
Temperature	i spike count	23	−0.21	0.34	-	-	-	-	-	-
Ganglion	i spike count	67	−0.22	0.08	-	-	-	-	-	-
i RMP	i spike count	67	−**0.40**	** < 0.01**	29	−**0.67**	** < 0.01**	21	0.40	0.07
i rebound spike count	i spike count	67	**0.49**	** < 0.01**	29	−0.13	0.51	21	-	-
i latency	i spike count	62	−**0.32**	**0.01**	28	0.11	0.59	21	−**0.63**	** < 0.01**
Δ RMP	Δ spike count	67	−**0.41**	** < 0.01**	29	−0.27	0.16	21	0.14	0.55
Δ rebound spike count	Δ spike count	67	−0.09	0.46	29	−0.16	0.42	21	-	-
Δ latency	Δ spike count	55	−0.02	0.89	28	−**0.60**	** < 0.01**	20	−0.30	0.20

**Wilcoxon tests**										

**x**	**y**	***n_1_/n_2_***	***Z***	***p***	***n_1_/n_2_***	***Z***	***p***	***n_1_/n_2_***	***Z***	***p***

i spike count IA	i spike count OR	44/23	0.96	0.34	-	-	-	-	-	-
Stim. i spike count	Cont. i spike count	23/19	−1.03	0.303	29/22	−0.53	0.596	21/22	−**4.61**	** < 0.001**
Stim. f spike count	Cont. f spike count	23/19	−0.25	0.800	29/22	−2.32	0.021	21/22	−**4.74**	** < 0.001**
Stim. Δ spike count	Cont. Δ spike count	23/19	−0.202	0.840	29/22	−0.23	0.819	21/22	1.89	0.058
Stim. Δ spike count	0	23	**3.87**	** < 0.001**	29	**4.37**	** < 0.001**	21	−**3.62**	** < 0.001**
Cont. Δ spike count	0	19	**3.67**	** < 0.001**	22	**3.95**	** < 0.001**	22	−**3.60**	** < 0.001**
Stim. i RMP	Cont. i RMP	23/19	−1.80	0.073	29/22	0.78	0.436	21/22	−**4.23**	** < 0.001**
Stim. f RMP	Cont. f RMP	23/19	−**3.41**	** < 0.001**	29/22	−2.19	0.029	21/22	−**3.90**	** < 0.001**
Stim. Δ RMP	Cont. Δ RMP	23/19	−**2.91**	**0.004**	29/22	−**2.94**	**0.003**	21/22	0.30	0.761
Stim. Δ RMP	0	23	−**3.74**	** < 0.001**	29	−**3.28**	** < 0.001**	21	−**3.67**	** < 0.001**
Cont. Δ RMP	0	19	−1.69	0.091	22	0.60	0.548	22	−**3.65**	** < 0.001**

*Statistically significant effects are marked in bold. i, initial values; f, final values after 10 min; Δ, changes within 10 min; n, number of recordings; r_s_, Spearman’s rank correlation coefficient; p, statistical p-value; z, value of the z-statistics; Stim., stimulus protocol; Cont., control protocol.*

**FIGURE 2:**
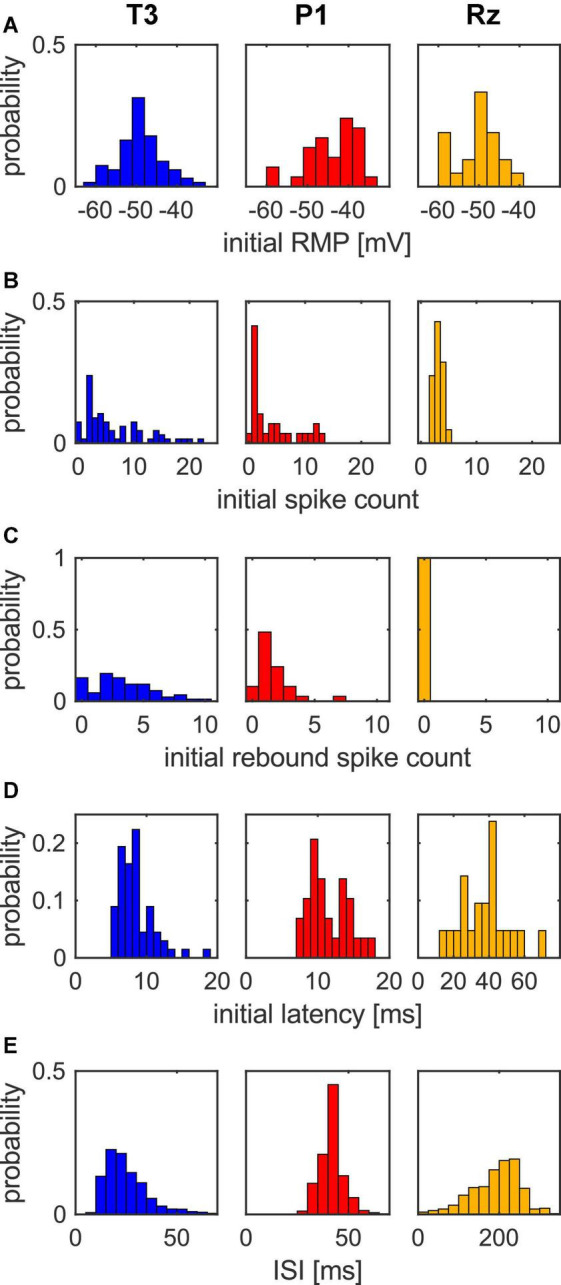
Distributions of response properties for T3 (*n* = 67), P1 (*n* = 29), and Rz (*n* = 21) cells in response to the stimulation protocol. **(A)** The initial RMP ranged from –65 to –35 mV for all cell types. **(B)** The initial spike count of T3 and P1 cells were variable, while all Rz cells fired consistently 3–5 spikes to +1 nA current. **(C)** Most T3 and P1 cells showed rebound spikes in trial 1, while Rz cells did not. **(D)** The initial first spike latency was characteristic for all cell types, with T3 cells having the shortest latencies and Rz cells the longest. Note that the x-axis-scaling is different for Rz. **(E)** ISI distribution over all trials. The distribution is skewed for T3 cells, while the ISI distribution of P1 cells is roughly symmetric around their preferred ISI of 42 ms. The distribution for Rz cells’ ISI is skewed, covering a large range. Note that the x-axis-scaling is different for Rz cells.

**FIGURE 3:**
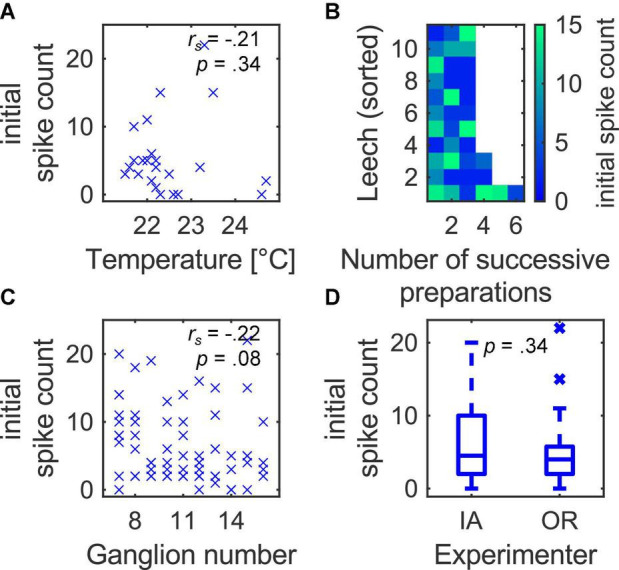
Confounder analysis for the initial spike count in T3 cells. **(A)** The temperature did not affect the initial spike count systematically. **(B)** The leech individual, sorted by the number of successive preparations, also did not reveal any obvious patterns in the initial spike count, indicating that the observed variability in the initial spike count was not systematically caused by the leech individual. **(C)** Neither the position of the midbody ganglion in the nerve cord, **(D)** nor the experimenter/setup had a significant effect on the initial spike count.

**FIGURE 4:**
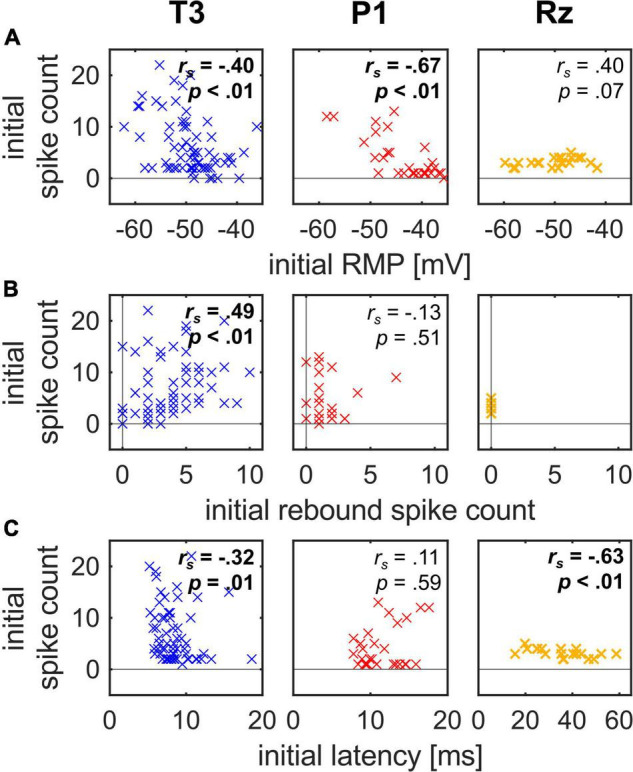
Interdependencies between initial response features in the stimulation protocol for all three cell types. Significant correlations are marked in bold. Sample sizes, correlation coefficients and *p*-values are given in Table 1. **(A)** The initial RMP correlated negatively with the initial spike count in T3 and P1 cells, but not in Rz cells. **(B)** The number of initial rebound spikes correlated positively with the initial spike count in T3 cells, but not in P1 cells. Rz cells did not show any rebound spikes. **(C)** The initial latency correlated negatively with the initial spike count in T3 and Rz cells, but not in P1 cells. Note that the few T3 and P1 cells with zero initial spikes by definition do not have a response latency and are therefore not included in these plots (see Table 1 for sample sizes).

**FIGURE 5:**
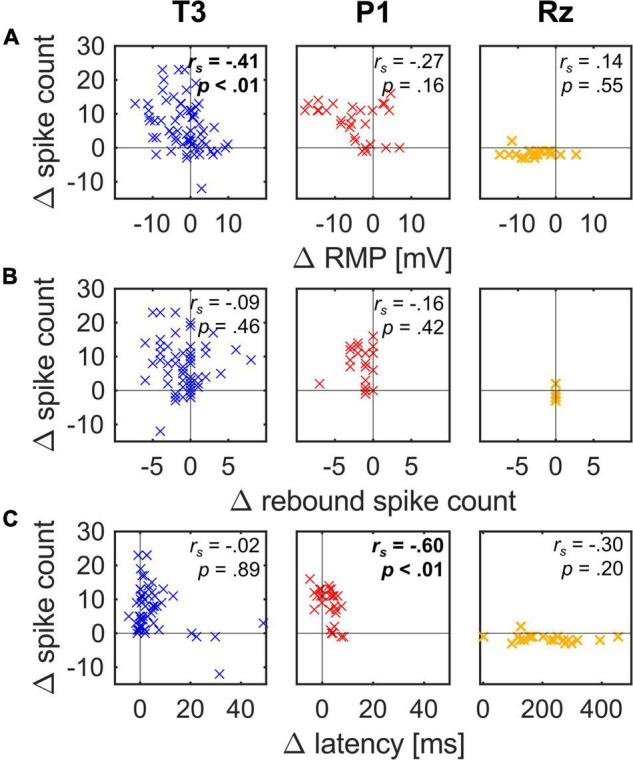
Interdependencies between changes (Δ) of response properties triggered by 10 min long repetition of the stimulation protocol for all three cell types. Significant correlations are marked in bold. Sample sizes, correlation coefficients and *p*-values are given in Table 1. **(A)** The Δ RMP correlated negatively with the Δ spike count in T3 cells. A similar tendency was visible (but not significant) in P1 cells but absent in Rz cells. **(B)** The Δ rebound spike count did not correlate with the Δ spike count in T3 and P1 cells. Rz cells did not show any rebound spikes. **(C)** The Δ latency correlates negatively with the Δ spike count in P1 cells, but not in T3 and Rz cells. Note that the x-axis-scaling is different for Rz cells.

**FIGURE 6:**
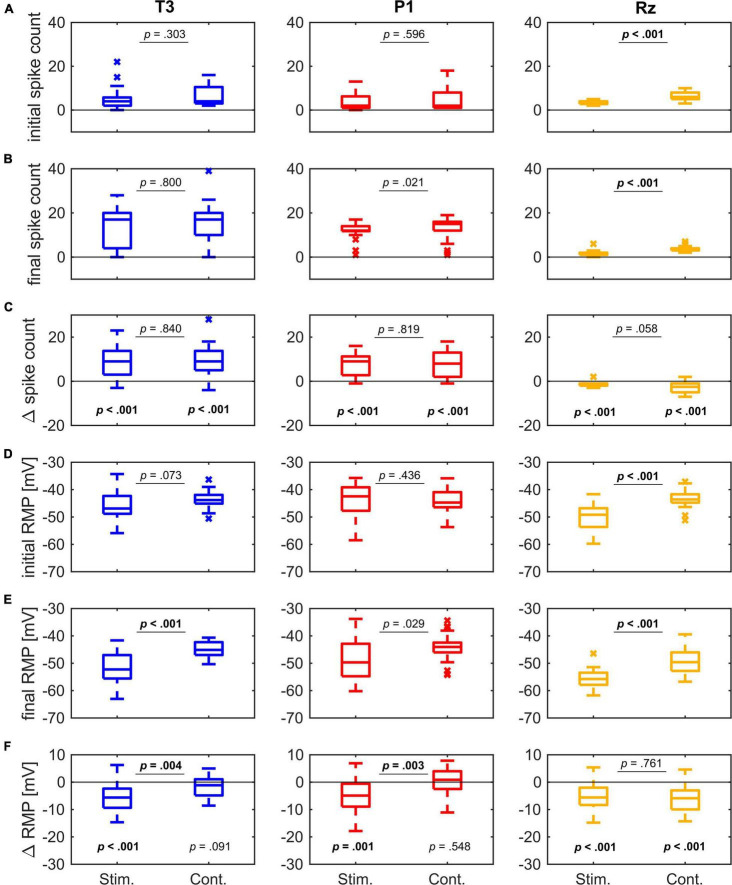
Spike count and RMP comparison between the stimulation protocol (Stim.) and the control protocol (Cont.) for all three cell types. Statistical test decisions are based on the Wilcoxon test. Multiple testing was accounted for by Bonferroni adjustment of the significance level from α < 0.05 to α’ < 0.01. Significant results are marked in bold**. (A)** The initial spike count did not differ between the two protocols for T3 and P1 cells. In Rz cells, the spike count was significantly higher in the control protocol. **(B)** The final spike count after 10 min did not differ between the two protocols in T3 and P1 cells, but in Rz cells. **(C)** The Δ spike count differed significantly from zero for all cell types and both protocols. T3 and P1 cells increased their spike count over time, irrespective of the protocol. Rz cells decreased their spike count over time. However, no difference was found between the two protocols for each of the cell types. **(D)** The initial RMP in the two protocols did not differ significantly for T3 and P1 cells. Rz cells were significantly more depolarized in the control protocol. **(E)** The final RMP after 10 min differed between the protocols for T3 cells, showing that cells in the stimulation protocol hyperpolarized significantly compared to cells in the control protocol. P1 cells showed the same tendency. Rz cells maintained their significant initial difference between the protocols. **(F)** The Δ RMP showed a significant hyperpolarization over time for T3 and P1 cells only in the stimulation protocol, but not in the control (see *p*-values at the panel bottom), leading to a significant difference between the responses to both protocols (see *p*-values at the panel top). Rz cells hyperpolarized in both protocols significantly, yielding no difference between the protocols.

## Results

We conducted intracellular single recordings from the mechanosensory T3 cells and P1 cells, as well as from one of the neuromodulatory Retzius cells. The stimulation protocol of 30 s consisted of pseudo-randomized 500 ms step currents varying in amplitude from −2 to +1.5 nA. It was repeated for 20 trials, leading to a recording time of 10 min. Figure 1 shows the stimulation protocol and one exemplary recording for each of the three cell types in trial 10 (Figure 1A), as well as the frequency-current (fI) curves for three representative cells per cell type in trial 1, 5, 10, 15, and 20 (Figures 1B–D). Insets within each graph depict the responses to the 1 nA current pulse in trial 1 and trial 20.

As expected, stronger input currents triggered more spikes, and the spike count saturated for very strong input currents in all trials and cell types. However, for T3 and P1 cells the number of spikes varied considerably between cells and over time. We found mechanosensory cells with constantly low spike counts (Figure 1B), cells that shifted from low to high spike counts (Figure 1C) and cells that had a high spike count from the beginning (Figure 1D). Moreover, almost irrespective of their initial spike count, the mechanosensory cells increased their activity over trials, while the Rz cells decreased it. More specifically, in response to a 1 nA current pulse, Rz cells quite consistently fired very similar numbers of spikes between neurons, but decreasingly over time. The three exemplary cells lowered their spike count from 2 to 1, from 2 to 1 and from 4 to 1, respectively (Figures 1B–D). On the other hand, the spike counts of the three exemplary T3 cells increased from 1 to 4, from 8 to 16 and from 15 to 24 spikes, respectively. The spike counts of the three exemplary P3 cells developed from 0 to 3, from 1 to 10, and the third P3 cell stayed at 12 spikes (see insets for trial 1 and trial 20). Most of the increase in T3 and P1 cell spike counts happened within the first 10 trials. After that, the spike count remained approximately constant (Figures 1B–D). To sum, T3 and P1 cells seem to have different spiking regimes between cells and over time, which appeared as shifts from phasic to tonic spiking in response to a long positive current input.

### T3 Cells and P1 Cells Spiked With a Precise Latency but With Variable Spike Counts, Rz Cells Responded With Consistent Spike Counts but With a Variable Latency

We collected a large dataset to further investigate and capture the initial variability in the response features elicited by the first presentation of the stimulation protocol. Figure 2 shows the distributions of the initial response features of resting membrane potential (RMP), spike count, rebound spike count, latency, as well as the inter-spike intervals (ISI) of all trials for T3 cells (*n* = 67), P1 cells (*n* = 29) and Rz cells (*n* = 21).

The initial RMP ranged from −65 to −35 mV for all cell types, with peaks around −50 mV for T3 cells and Rz cells, and at around −40 mV for P1 cells (Figure 2A). The initial input resistance ranged from 6 to 80 MΩ for all cell types with a median of 30 MΩ for T3 cells, 20 MΩ for P1 cells and 18 MΩ for Rz cells (data not shown).

The initial number of spikes in response to the 500 ms long 1 nA current pulse varied for T3 cells, ranging from 0 to 23 spikes, with *CV* = 0.86. For P1 cells, spike counts ranged from 0 to 12, with *CV* = 0.98. For both cell types, the spike count distribution was strongly skewed with a peak at 2–3 spikes and a long tail to higher spike counts. In contrast, all recorded Rz cells consistently spiked 2–5 times, with *CV* = 0.27 (Figure 2B).

Another clear difference is that the mechanoreceptor cells typically fired rebound spikes after the −2 nA current pulse in the stimulation protocol, while Rz cells never showed rebound spikes. Most P1 cells fired exactly 1 rebound spike, some up to 6, while T3 cells again exhibited a broader distribution with up to 10 initial rebound spikes in some cases (Figure 2C).

The distributions of initial response latencies revealed characteristic peaks for each of the cell types. T3 cells had a median latency of 8.1 ms (*CV* = 0.29), P1 cells of 10.8 ms (*CV* = 0.24) and Rz cells of 36.3 ms (*CV* = 0.35). T3 cells started their spike response quite precisely within 5–10 ms and P1 cells within 7–15 ms after stimulus onset. In contrast, Rz cells started spiking with a rather random latency, covering a broad range from 10 to 70 ms (Figure 2D). These differences between cell types cannot be explained by different numbers of spikes, since the fraction of T3 and P1 cells that fired 4 or less spikes (like Rz cells) still had very short median latencies of 8.4 and 10.5 ms, respectively (Supplementary Figure 1).

The differences in spike timing between the cell types also became apparent from the ISI distribution. Note that the ISI distributions shown in Figure 2E are not restricted to the initial response, but the ISIs in response to the 1 nA current pulse of all trials were pooled to derive a meaningful distribution. Corresponding to their large spike counts and their tendency to cease spiking before the end of a current pulse, T3 cells had the shortest median ISI of 23 ms, and the highest *CV* of 0.64. The skewed distribution with occasional ISIs of up to 60 ms can be explained by the typical response pattern of T3 cells, with ISIs being shortest at the beginning of a current pulse and getting longer with every spike (see Figure 1 for examples). P1 cells, on the other hand, yielded a rather narrow, symmetric ISI distribution with a median ISI of 42 ms and a low *CV* of 0.14, indicating their rhythmic and precise spiking (see Figure 1 for examples). This is one of the most prominent distinctions between the two mechanosensory cell types. The median ISI for Rz cells lied much higher at around 200 ms, with their distribution covering a very broad range from 15 to 300 ms and a *CV* of 0.30. These findings reflect that Rz cell spikes could occur at any times during the stimulation, while P1 cells fired rhythmically, and T3 cells mainly responded to stimulus onset.

In conclusion, T3 cells and P1 cells spiked variably in terms of their numbers of spikes but started their responses temporally precisely. Rz cells, on the other hand, were consistent in their numbers of spikes, but imprecisely in their latencies.

### The Variability in the Initial Spike Count Was Not Caused by an Experimental Confounder

Apart from the differences between cell types, the variability of the initial spike count within T3 cells was particularly striking. To confirm that this high variability is cell-type-specific rather than an experimental artifact, we investigated the influence of the potential confounders *temperature*, *leech ID*, *ganglion number*, and *experimenter*/*setup* on the initial spike count in T3 cells (Figure 3). This analysis was restricted to only one cell type, because our data set did not include temperature measurements and data from two experimenters for P1 and Rz cells.

The temperature measured in the petri dish was constant during each experiment (data not shown) but varied from 21.5 to 24.6°C between experiments. Within this range, temperature did not affect the initial spike count significantly (Figure 3A; *n* = 23, *r*_*s*_ = −0.21, *p* = 0.34).

We next visualized the initial spike count to check for interindividual differences between leeches. Figure 3B shows the heat map for the initial spike count of *n* = 38 recordings in 11 leeches, from which three or more preparations were included in this study. Every row comprises the experiments accomplished in the same leech, and rows are sorted by the numbers of the successive preparations taken from their nerve cords, which are shown in the columns. The heat map does not reveal a pattern. The absence of uniformly colored rows means that in each leech, both high and low initial spike counts were observed. Hence, the variability in the initial spike count did not originate from leech individuals. Moreover, there is also no color pattern in the columns, meaning that both low and high spike counts could be obtained in the first, as well as in the last preparation performed on the same day.

Since all experiments were carried out on isolated midbody ganglia, we also investigated if the position of the ganglion in the nerve cord influenced the initial spike count in T3 cells. The ganglion position ranging from 7 to 16 (counted from anterior) did not affect the initial spike count (Figure 3C; *n* = 67, *r*_*s*_ = −0.22, *p* = 0.08).

Moreover, the dataset was recorded by two experimenters (IA & OR) in two separate experimental setups. We therefore tested if slight changes in the equipment or the handling of the preparations could have caused the variability in the initial spike count. This was not the case (Figure 3D; *z* = 0.96, *p* = 0.33). In conclusion, none of the four potential confounders caused the observed variability in the initial spike count of T3 cells.

### The Spike Count of T3 Cells and P1 Cells Correlated Negatively With the Initial RMP

Next, we investigated if the initial variability in spike count correlated with the RMP, the number of rebound spike count or the latency (Figure 4). Correlation coefficients and *p*-values are given in Table 1.

The initial RMP correlated negatively with the initial spike count for T3 cells and P1 cells (Figure 4A; T3 cell: *n* = 67, *r*_*s*_ = −0.40, *p* < 0.01; P1 cell: *n* = 29, *r*_*s*_ = −0.67, *p* < 0.01). That is, the more hyperpolarized these cells were, the larger number of spikes they generated. This finding is consistent with the results reported by Meiser et al., 2019 that T cells increase their spike count with hyperpolarization. However, it is contrary to standard expectations that a more hyperpolarized cell is further away from the spike threshold and therefore fires fewer spikes. Rz cells showed a tendency in this expected direction, but the dependency between the resting membrane potential and the spike count was not significant.

The initial number of rebound spikes correlated positively with the initial spike count for T3 cells (Figure 4B; *n* = 67, *r*_*s*_ = 0.49, *p* < 0.01). That is, the more spikes a T3 cell fired in response to the 1 nA current pulse, the more rebound spikes were generated. This finding suggests that some cells are more excitable in general. This relationship was not significant for P1 cells, most of which elicited exactly one rebound spike. And this could obviously not apply to Rz cells, which do not have any rebound spikes.

The initial latency correlated negatively with the initial spike count for T3 and Rz cells (Figure 4C; T3: *n* = 62, *r*_*s*_ = −0.32, *p* = 0.01; Rz: *n* = 21, *r*_*s*_ = −0.63, *p* < 0.01). The more spikes T3 and Rz cells elicited, the shorter their initial latency was.

In conclusion, the initial RMP correlated negatively with the highly variable initial spike count in T3 cells and P1 cells, which is contrary to the commonly expected higher spike counts for more depolarized cells. The T3 cells additionally showed a positive correlation of the initial spike count with the initial rebound spike count and a negative correlation with the initial latency, suggesting that some of the cells in the sample were generally more excitable than others. The response properties of Rz cells are again quite different from T3 and P1 cell responses, because of their low variability in initial spike count.

### The Spike Count of T3 Cells and P1 Cells Increased Over Time, While Rz Cell Activity Decreased

Figure 1 suggested not only that the initial spike count was variable in T3 cells and P1 cells, but also that their spike count systematically increased over time. To further examine these observations, we determined the relationships between the changes in the response features over time, namely Δ RMP, Δ rebound spike count, Δ latency, and Δ spike count. Δ was defined as the difference of a response feature observed after 10 min of recording time (trial 20 for *stim*, trial 3 for *cont*) minus the initial response (trial 1). Correlation coefficients and *p*-values are given in Table 1.

Most T3 cells and P1 cells increased their spike count after 10 min of stimulation (y-axes in Figure 5A, T3 cell: median = 5; P1 cell: median = 9). This increase was variable, where some cells increased their spike count in response to the 1 nA current pulse considerably. In some T3 and P1 cells, the final spike counts were approximately seven times larger than the initial values, leading to up to 23 spikes in T3 and 16 spikes in P1 cells. The spike count decreased over time only in 10 out of 67 T3 cells (15%), and 2 out of 29 P1 cells (7%) (also see y-axes in Figure 5A). Rz cells, on the other hand, consistently decreased their spike count, with only one exception (median = −2).

Most T3, P1 and Rz cells hyperpolarized over time (Figure 5A, T3 cell: median = −0.77 mV, P1 cell: median = −4.90 mV, Rz cell: median = −5.59 mV, the RMP after 10 min was corrected for the electrode offset drift). In T3 cells, the changes in RMP correlated negatively with the changes in spike count (Figure 5A; *n* = 67, *r*_*s*_ = −0.41, *p* < 0.01). That is, the more the RMP hyperpolarized over time, the more strongly the spike count increased. This finding is consistent with the negative correlation between the initial values of spike count and RMP (Figure 4A), and with Meiser et al., 2019. We observed the same tendency in P1 cells, even though this relationship was not significant. The decreasing spike count in Rz cells did not correlate with the change in RMP.

Most T3 cells and P1 cells tended to decrease their number of rebound spikes over time (Figure 5B). Only 11 of the 67 T3 cells (16%) increased their rebound spike count, and not a single P1 cell did so. We did not find any significant correlations between the changes in rebound spike count and in spike count.

Lastly, most T3 cells and P1 cells and all Rz cells increased their latency over the course of 10 min. However, only in P1 cells did the changes in latency correlate negatively with the changes in spike count (Figure 5C; *n* = 28, *r*_*s*_ = −0.60, *p* < 0.01), which might be due to the more regular spiking pattern of this cell type compared to T3 and Rz cells.

In conclusion, even though all three cell types hyperpolarized within the course of 10 min, their spike count developed differently over time. While T3 and P1 cells increased their spike count, Rz cells did the opposite. In accordance with Meiser et al., 2019, we found that the more the RMP of T3 cells hyperpolarized, the more these cells spiked. However, this negative correlation between the changes in RMP and spike count was only significant for T3 cells, but not for P1 cells.

### The Increase in Spike Count Was Not Activity-Dependent

To investigate if the increase in spike count and the decrease in RMP is activity-dependent, as suggested by Meiser et al., 2019, we also recorded all three cell types with a control protocol that triggered as few spikes as possible. The protocol consisted of only one 500 ms long 1 nA pulse, followed by 5 min without stimulation. Hence, in contrast to the rather unphysiological situation of cells being exposed to repeated strong current injections during the stimulation protocol, these control experiments do not manipulate the cells beyond the penetration of the membrane with an extracellular electrode and a single test pulse. Within the period of zero stimulation, Rz cells showed spontaneous spiking of typically initially 1–2 Hz which decreased over time. Some of the T3 cells spiked occasionally and P1 cells did not fire action potentials during these extended periods without stimulation. We saw inhibitory inputs in the majority of the T3 cells and also excitatory postsynaptic potentials in some T3 cells, while no synaptic potentials were present in P1 cells (Supplementary Figure 2).

We compared the initial values and time-dependent changes in RMP and spike count of the stimulation protocol with the control protocol for all three cell types (Figure 6). In this figure only experiments performed by OR are included to ensure similar sample sizes and maximal comparability between the experiments performed with the two protocols. Note that due to multiple testing, we corrected the significance level from α = 0.05 to α’ = 0.01 for all Wilcoxon tests displayed in Figure 6.

The spike count did not differ between the two protocols in T3 cells and P1 cells, but Rz cells spiked more often in the control protocol, both in the initial responses (Figure 6A), as well as after 10 min of recording time (Figure 6B). Spike counts changed significantly in all cell types and both protocols (Figure 6C), with Rz cells decreasing and the mechanoreceptors increasing their spike counts. For the stimulation protocol, 20 of 23 T3 cells (87%) and 24 of 29 P1 cells (83%) increased their spike count after 10 min. For the control protocol, the same percentage of cells (T3 cells: 17 of 19 cells, 89% and P1 cells: 20 of 22, 91%) increased their spike count in the same period. Since the changes in spike counts were not significantly different between both protocols for any of the cell types, we can conclude that the change of excitability does not depend on repeated stimulation.

The initial RMP did not differ between the two protocols in T3 and P1 cells, but Rz cells were more depolarized in the control protocol (Figure 6D). During 10 min of recording time, the RMP hyperpolarized in T3 and P1 cells only in response to the stimulation protocol, but not in the control recordings (Figure 6F). Consequently, the final RMP was significantly different between the two protocols in T3 cells, and P1 cells showed the same trend (Figure 6E). In contrast, Rz cells hyperpolarized significantly over time in both protocols (Figure 6F) and maintained their initial differences (Figure 6E).

In conclusion, both protocols led to a similar increase in spike count in the mechanoreceptors (Figure 6C). This finding suggests that the increase in spike count over time is not activity-dependent but occurs also when no spikes are elicited for a long period of several minutes. Moreover, the membrane potential of the mechanoreceptors only hyperpolarized significantly when they were stimulated repeatedly (Figure 6F). Hence, the increase in excitability also did not require the hyperpolarization of the resting membrane potential. These findings challenge the mechanism of short-term intrinsic plasticity suggested by Meiser et al., 2019, which assumed spiking activity and subsequent hyperpolarization as the starting point of the cell-intrinsic activity changes.

## Discussion

In this study, we showed how variable neuronal response features in three different cell types of the leech are—over time and between cells. We found that the mechanosensory T3 and P1 cells, despite their differences in temporal response patterns, share several properties that differ considerably from the response behavior of Rz cells. In the mechanosensory cells, the initial spike counts are very variable, while the latency is precise across the samples of both cell types. In contrast, Rz cells consistently fire only few action potentials, but the timing of their first spike is rather random.

The main difference between the mechanosensory cells and the Rz cells, however, becomes evident after several minutes of recording. Even though the membrane potential of all three cell types hyperpolarizes during repeated current stimulation, only the Rz cell decreases the number of generated spikes, as it would be expected from the resting potential moving away from the spike threshold. In contrast, the number of spikes increased in T3 cells with repeated current injection, as was already shown in our previous study (Meiser et al., 2019). We observed the same counterintuitive effect of increasing spike counts for hyperpolarized P1 cells, emphasizing the similarity of the two mechanoreceptors.

In a control experiment, we demonstrated that this time-dependent increase in excitability does not depend on previous spiking activity or unphysiologically high current injections. In the absence of current stimulation, when no spikes are elicited for several minutes, the resting membrane potential does not hyperpolarize significantly, but the excitability of T3 and P1 cells still increases.

### Initial Variability Between Cell-Types and Between Cells of the Same Type

The differences in the responses of T, P and Rz cells are well known from decades of research on the leech nervous system (Nicholls and Baylor, 1968; Muller and Scott, 1981; Kristan et al., 2005; Wagenaar, 2015). T and P cells are both mechanosensory neurons detecting sensory input with their extended processes in the skin (Nicholls and Baylor, 1968; Pinato and Torre, 2000). The two cell types respond with distinct sensitivities to tactile input and their action potentials differ in duration and amplitude (Nicholls and Baylor, 1968). Rz cells, on the other hand, are neurosecretory cells, releasing serotonin into the ganglion and thereby affecting a variety of other neurons and tissues (Lent, 1973; Leake, 1986). The two Rz cells in a ganglion are strongly electrically coupled (Welzel and Schuster, 2018), spontaneously active (Lent, 1977) and can autoregulate themselves *via* serotonin (Acosta-Urquidi et al., 1989; Calviño and Szczupak, 2008).

These different functions of T, P and Rz cells manifest themselves in different ion channels underlying their distinct response behavior (Johansen, 1991; Kleinhaus and Angstadt, 1995). All three cell types possess Na^+^, K^+^ and Ca^2+^ currents (Stewart et al., 1989), as well as hyperpolarization-activated (I_*h*_) currents, which are most prominent in P cells (Gerard et al., 2012). However, T and P cells differ from Rz cells by not expressing A-type K^+^ currents (I_*A)*_ (Stewart et al., 1989), and by expressing Na^+^ channels that can be blocked by TTX (Kleinhaus and Prichard, 1976, 1983).

In addition to the biophysical properties, also the network interactions differ between the cell types. Despite being mechanoreceptors that provide information on sensory input to the network, T cells are known to also receive synaptic inputs. The three bilateral pairs of T cells in one ganglion are coupled with each other (Nicholls and Baylor, 1968; Baylor and Nicholls, 1969b; Gu et al., 1989; Li and Burrell, 2008). T cells also receive inhibitory and excitatory synaptic input from P and N cells *via* polysynaptic connections (Burgin and Szczupak, 2003), as well as reafferent inhibition from the motor system during crawling (Alonso et al., 2020). Furthermore, several interneurons are known to be connected with T cells *via* electrical synapses, many of which can be seen in neurobiotin stainings (Kretzberg et al., 2016; Segura et al., 2020), and some of which are rectifying (Firme et al., 2012). In our dataset, the effect of the network on the T3 cell was clearly visible as postsynaptic potentials during the zero stimulation periods in the repeated control protocol (Supplementary Figure 2). P cells also display neurobiotin coupling *via* gap junctions with several interneurons (Segura et al., 2020), but we did not see any postsynaptic potentials in our P1 cell recordings. Rz cells are only coupled to their homolog Rz cell in the same ganglion (Segura et al., 2020).

Besides the previously known differences between the cell types, we found considerable variability between the initial responses of individual cells. Our confounder analysis revealed that none of the four factors temperature, leech, ganglion, or experimenter/setup can explain this variability. Hence, we conclude that the variability in our data represents physiological differences between the cells, rather than experimental issues. The individual differences in response features between cells of the same type could be due to individually different ion channel equipment, as it was found in several vertebrate and invertebrate systems (Golowasch et al., 2002; Prinz et al., 2004; Schulz et al., 2006; Günay et al., 2008). Also, differences in synaptic conductances could lead to individual variation, as it was shown for the leech heartbeat circuit (Calabrese et al., 2011).

For the Rz cells, a striking aspect of variability was the unexpected significant difference in the initial responses between the two protocols (Figures 6A,B). It is possible that the 19 s of electrical pulses applied in the stimulation protocol before the 1 nA pulse (see Figure 1A) already influenced the response features during the first trial. However, the resting membrane potential during the first second of the recording before any current stimulus was applied, was already significantly different between the two samples of Rz cells in experiments with the stimulation and the control protocol (data not shown). Additionally, the RMP in the first second of the recording did not differ significantly from the RMP at second 18–19—neither for the stimulation protocol, nor for the control protocol. Hence, the difference in RMP and probably also spike counts cannot be explained by the differences in the electrical stimulation protocols. It is possible that the observed significant differences are just false positives. However, there could also be systematic differences in the samples, because the two data sets were collected from different batches of leeches. The serotonin-releasing Rz cells are known to have autoreceptors, modulating response features and probably also the further serotonin release (Calviño and Szczupak, 2008). Since seasonal variations of the endogenous serotonin content were reported for the leech (Catarsi et al., 1990), it could have had an impact that the control experiments were recorded between April and June, and the stimulation experiments between August and September.

For the mechanoreceptors, a specific factor adding to the variability might have been a mix of different functional cell subtypes. Even though all of the cells included in this study were recorded at the same anatomical position of T3 and P1 cells, it is known that the subtypes of the mechanoreceptors can switch the positions of their receptive fields (Kretzberg et al., 2016). Many T3 cells have their receptive fields ventrally, but the remaining individuals with a dorsal or a lateral receptive field might have systematically different response features due to their anatomical structure, synaptic partners, or channel distribution. Future experiments combining electrophysiological and anatomical characterization with dye-filled intracellular electrodes are needed to reveal if electrophysiological and anatomical differences correlate.

### Response Features of Individual Cells Change Over Time

We showed how drastically individual cells can change their neuronal response features over time and that these effects are fundamentally different between cell types. In the study of Meiser et al., 2019, it was suggested that repeated stimulation leads to increased spiking and therefore to Na^+^ influx, which by activating the Na^+^/K^+^ pump slowly hyperpolarizes the resting membrane potential. This hyperpolarization in turn was hypothesized to close putative slow, voltage-dependent K^+^-channels, mitigating K^+^ efflux and therefore causing increased spiking in response to a given current pulse over time (Meiser et al., 2019).

In this study, repeated somatic stimulation hyperpolarized the RMP in all three cell types with the stimulation protocol, but not with the control protocol (Figure 6). That is, repeated stimulation could indeed increase Na^+^/K^+^ pump activity, which in turn hyperpolarizes the membrane potential, as it has been shown in T cells for both somatic (Jansen and Nicholls, 1973; Scuri et al., 2007) and tactile stimulation (Scuri et al., 2002). On the other hand, we clearly showed that these changes in the resting membrane potential are not necessary to increase the spike count over time (Figure 6). This finding questions the suggested involvement of slow voltage-dependent K^+^-channels, which could be analyzed in future experiments by pharmacological blocking e.g., with XE-991, as it was previously done in drosophila and C. elegans (Wei et al., 2005; Cavaliere and Hodge, 2011).

An alternative explanation of the excitability changes over time could be the involvement of hyperpolarization-activated (I_h_) currents. I_h_ is mediated by HCN channels and was found in many leech neurons, including T, P and Rz cells (Gerard et al., 2012; Angstadt et al., 2021). Their activation range of −65 to −100 mV (50–100% opening probability) and their relatively long activation time constant of a few hundreds of milliseconds causes a voltage sag during hyperpolarization of the RMP induced by negative current injection (Gerard et al., 2012). Our recordings showed this characteristic feature in the responses of all three cell types, most prominently in P cells. In general, I_h_ causes Na^2+^ and K^+^ influx to the cell, thereby acting as homeostatic mechanisms to rebalance the membrane potential (Magee, 1999). Moreover, it was shown that I_h_ can shunt excitatory postsynaptic potentials, and therefore can decrease excitability (Magee, 1998). Accordingly, a possible run-down of HCN channels over time could have the effect we see in our mechanoreceptor recordings: the RMP hyperpolarizes, while the excitability increases. We tested this idea by calculating the voltage sag ratio for all trials of all cell types and protocols (Supplementary Figure 3). Since we did not find systematic decrease of the voltage sag ratio over trials in any of the studied cell types, we conclude that the increase in excitability is not caused by the run-down of HCN channels over time.

Beyond the presented evidence that slow voltage-dependent K^+^-channels and HCN channels both are unlikely candidates for explaining the systematic increase in spike count over time in T3 and P1 cells, we can only speculate about other physiologically relevant causes. Two conceivable and omnipresent actors are voltage-gated calcium channels (VGCCs) and serotonin. Both could have excitatory effects on the spike count in T and P cells (Belardetti et al., 1984; Catarsi and Brunelli, 1991b; Gascoigne and McVean, 1991; Dierkes et al., 2004). Additional pharmacological experiments would be needed to investigate their role in short-term intrinsic plasticity.

However, also physiologically irrelevant experimental artifacts need to be considered as a possible source of the observed changes in excitability. Even though we excluded some potential experimental confounders (Figure 3), there are at least two uncontrolled experimental artifacts that could have impacted our results. The first critical artifact in intracellular recordings is the rupturing of the cell membrane by the electrode. This causes ion leakage from the intracellular to the extracellular space and vice versa, possibly leading to a depolarization of the RMP. Over time, the lipid bilayer of the membrane seals the gap around the tip of the electrode. The resting membrane potential can repolarize and the cell spikes “normally” again. If the effect of membrane rupturing varies between experiments, this could explain the variability in the initial RMP in all three cell types (Figure 2A). However, it could not explain the fundamentally different development of the spiking behavior of the mechanosensory cell types versus the Rz cell.

The second experimental artifact could be cell dialysis. Hooper et al. (2015) showed in lobster and leech neurons that electrode-fill solutions with much higher K^+^ concentrations than the neuron cytoplasm (∼250 mM) can cause non-physiological changes in neuron properties over time by K^+^ of the electrode solution leaking into the cell. Among the changes reported in their study was a significant hyperpolarization of the RMP in lobster neurons, but not in leech Rz cells when using electrode fills of 4 M KAc Hooper et al. (2015). We tested the effect of cell dialysis with a more cytoplasmic electrode-fill solution of 300 mM. These experiments still led to a comparable increase in spike count in T3 cells (*n* = 12, data not shown), making cell dialysis a rather improbable explanation for the observed time-dependent changes in neuronal response features.

### Conclusion

We could show that the cell-type-specific changes in excitability in prolonged recordings can neither be explained by slow, voltage dependent K^+^ channels that would require hyperpolarization to trigger the effect, nor by the run-down of HCN channels, or any of several considered potential experimental artifacts. Hence, we suggest that mechanosensory cells, but not Rz cells, can shift between different spiking regimes. The recorded cells distributed in different regimes at the start of the recording could explain the variability in the initial responses. A prolonged recording time seems to shift the probability for the cells to be in a more responsive spiking regime, leading to the increase in spike count over time. In the intact animal, we would expect a fraction of these cells to also shift their spiking regimes back to fewer spikes. However, in our intracellular recordings, we find a decrease in excitability only for very few T3 and P1 cells, even in the absence of current stimulation. Hence, it appears that the mere intracellular recording situation might cause enduring perturbations to cellular response features during prolonged recordings.

Both types of stimulation used in this study can hardly be compared with the natural condition of a living leech, constantly receiving tactile input to the skin that leads to extensive network activity (Fathiazar et al., 2018). In such a situation, we expect the membrane potential at the T cell soma to be impacted both by the action potentials elicited by touch to the skin’s receptive fields, as well as by synaptic inputs from various interneurons. Maybe the unnatural situation in an isolated neuron with missing reafferent synaptic inputs during behavior (Alonso et al., 2020), and the disruption of mechanisms like conduction block (Nagy et al., 1981; Macagno et al., 1987; Mar and Drapeau, 1996; Baccus et al., 2000) and the interaction of spike initiation zones (Calabrese and Kennedy, 1974; O’Shea, 1975; Calabrese, 1980; Städele and Stein, 2016) contributes to the shift in excitability and the high variability between neurons of the same type. Hence, the results of our systematic comparison of responses without stimulation to very strong electrical stimulation in isolated ganglia, need to be checked with experiments in semi-intact preparations under more naturalistic conditions. It remains to be investigated if shifts between different spiking regimes play a role in the context of behavioral reactions to touch stimuli. If they do, the apparent “randomness” in the mechanoreceptor responses could be the key to understanding what makes a nervous system flexible and robust to changes.

## Data Availability Statement

The original contributions presented in the study are included in the article/Supplementary Material. The datasets generated for this study are publicly available at https://gin.g-node.org/JSS/Scherer_et_al_2022.

## Author Contributions

JSS, SM, and JK: conceptualization and interpretation. IA and OR: data collection. JSS: data analysis. JK: project administration and supervision. JSS and JK: drafting manuscript. All authors contributed to the article and approved the submitted version.

## Conflict of Interest

The authors declare that the research was conducted in the absence of any commercial or financial relationships that could be construed as a potential conflict of interest.

## Publisher’s Note

All claims expressed in this article are solely those of the authors and do not necessarily represent those of their affiliated organizations, or those of the publisher, the editors and the reviewers. Any product that may be evaluated in this article, or claim that may be made by its manufacturer, is not guaranteed or endorsed by the publisher.
