# Supplementary figures and images for: Initial Variability and Time-Dependent Changes of Neuronal Response Features Are Cell-Type-Specific
Source: Front Cell Neurosci. 2022 Apr 27;16:858221. doi: 10.3389/fncel.2022.858221 (PMC9092978; doi:10.3389/fncel.2022.858221)

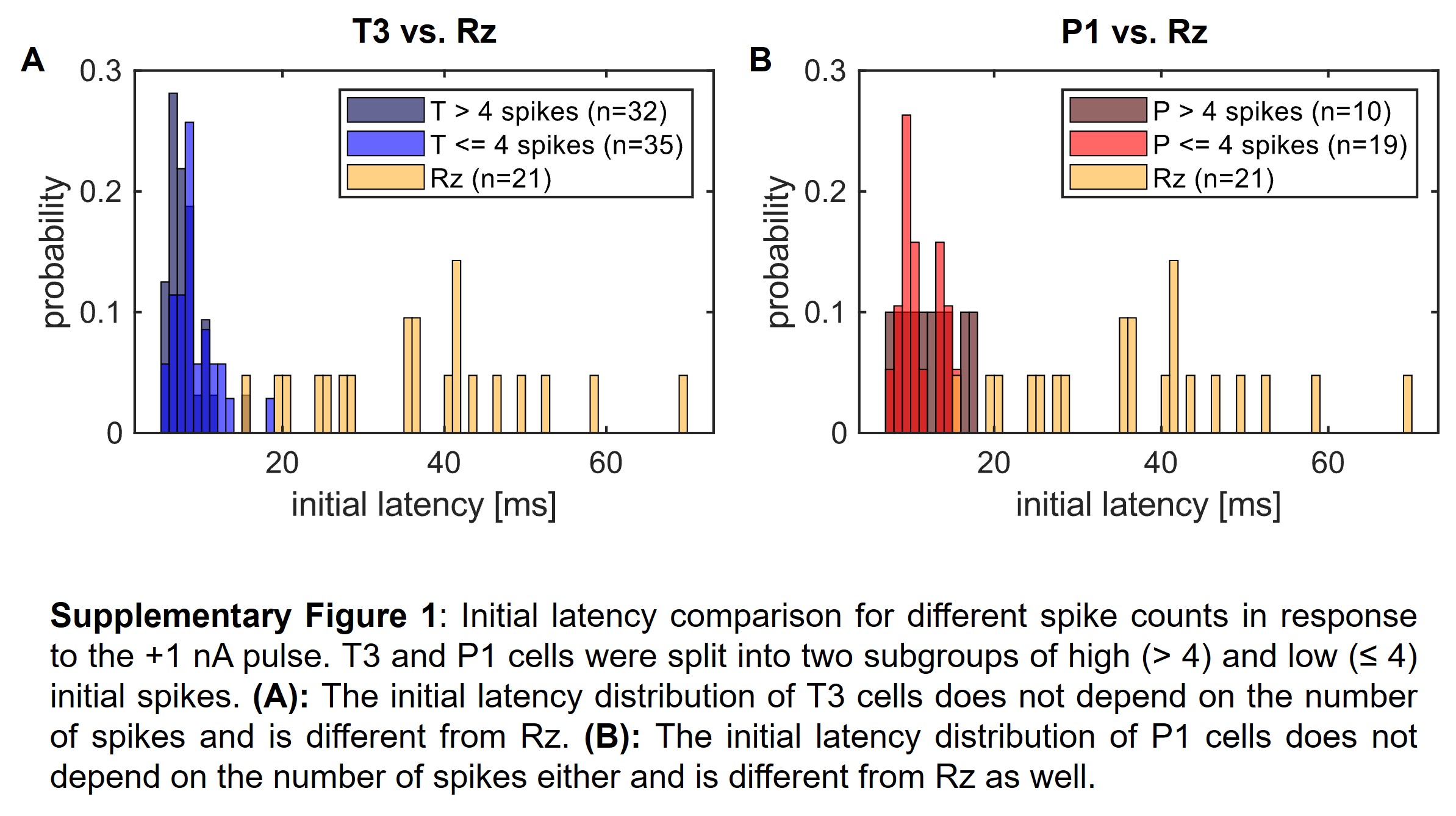

Supplement: Supplementary file 1 [file Image_1.JPEG]

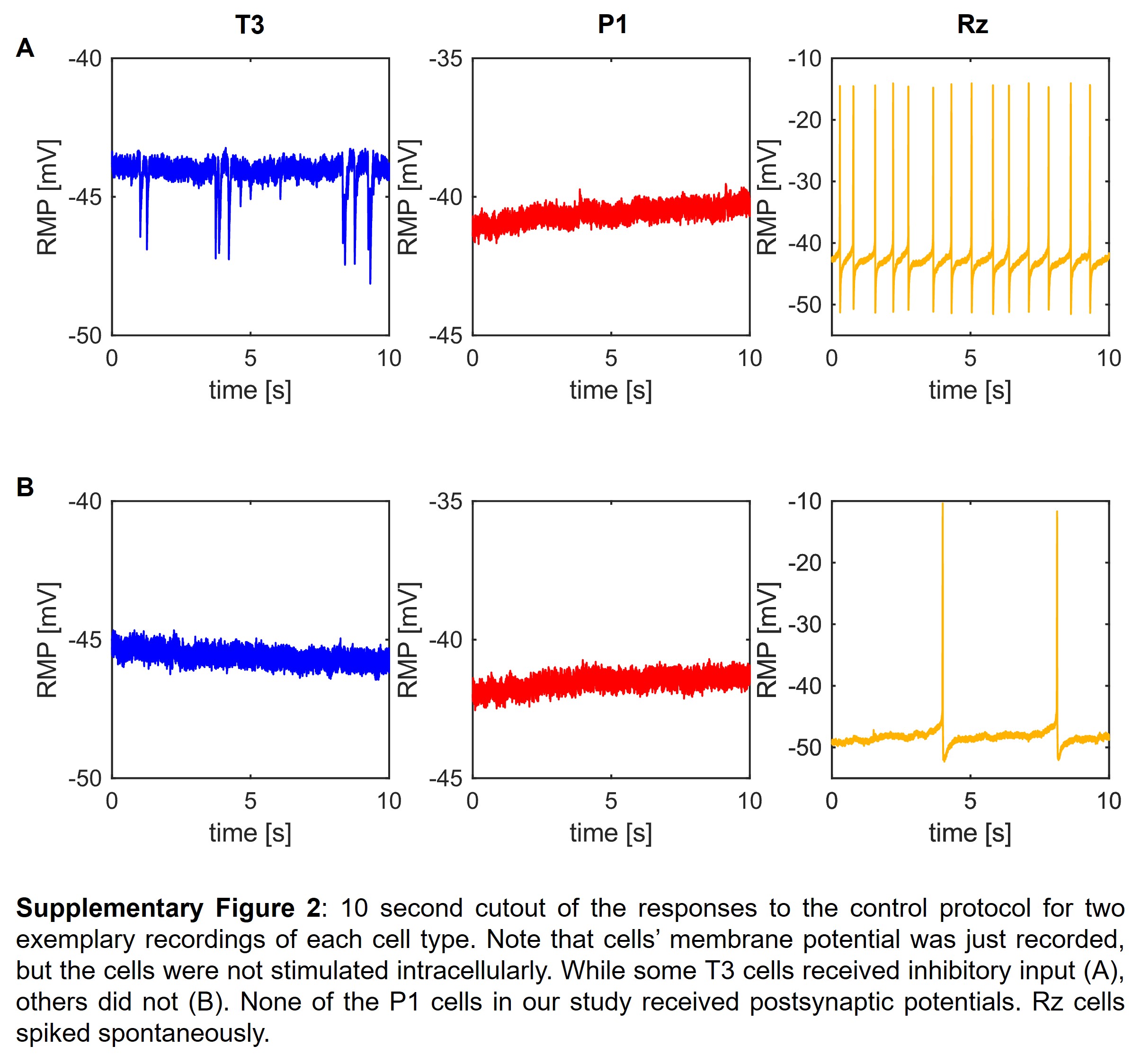

Supplement: Supplementary file 2 [file Image_2.JPEG]

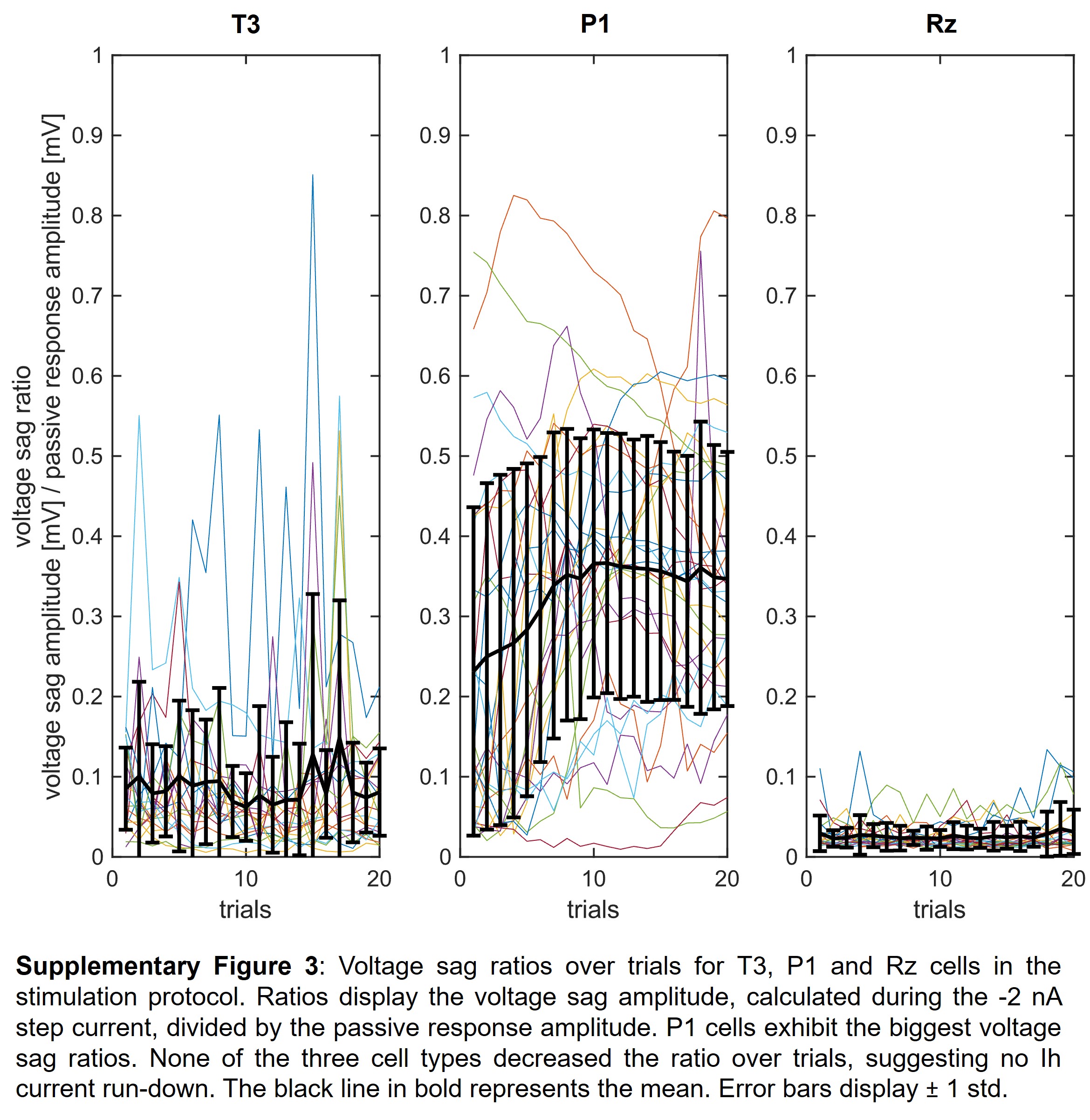

Supplement: Supplementary file 3 [file Image_3.JPEG]
